# Methylotrophic Communities Associated with a Greenland Ice Sheet Methane Release Hotspot

**DOI:** 10.1007/s00248-023-02302-x

**Published:** 2023-10-16

**Authors:** Matěj Znamínko, Lukáš Falteisek, Kristýna Vrbická, Petra Klímová, Jesper R. Christiansen, Christian J. Jørgensen, Marek Stibal

**Affiliations:** 1 Department of Ecology, Faculty of Science, Charles University, Prague, Czechia; 2 Current address: Department of Environmental and Biological Sciences, University of Eastern Finland, Kuopio, Finland; 3 Department of Geoscience and Natural Resource Management, University of Copenhagen, Copenhagen, Denmark; 4 Department of Ecoscience, Arctic Environment, Aarhus University, Roskilde, Denmark

**Keywords:** Methylotrophs, Methanotrophs, Subglacial environment, Greenland Ice Sheet

## Abstract

Subglacial environments provide conditions suitable for the microbial production of methane, an important greenhouse gas, which can be released from beneath the ice as a result of glacial melting. High gaseous methane emissions have recently been discovered at Russell Glacier, an outlet of the southwestern margin of the Greenland Ice Sheet, acting not only as a potential climate amplifier but also as a substrate for methane consuming microorganisms. Here, we describe the composition of the microbial assemblage exported in meltwater from the methane release hotspot at Russell Glacier and its changes over the melt season and as it travels downstream. We found that a substantial part (relative abundance 27.2% across the whole dataset) of the exported assemblage was made up of methylotrophs and that the relative abundance of methylotrophs increased as the melt season progressed, likely due to the seasonal development of the glacial drainage system. The methylotrophs were dominated by representatives of type I methanotrophs from the Gammaproteobacteria; however, their relative abundance decreased with increasing distance from the ice margin at the expense of type II methanotrophs and/or methylotrophs from the Alphaproteobacteria and Betaproteobacteria. Our results show that subglacial methane release hotspot sites can be colonized by microorganisms that can potentially reduce methane emissions.

**Supplementary Information:**

The online version contains supplementary material available at 10.1007/s00248-023-02302-x.

## Introduction

Subglacial environments, situated at the interface between glaciers and ice sheets and their bedrock and/or overlying sediments, contain large reserves of organic matter (OM), overridden during periods of glacial advance [1, 2]. Microbial oxidation of this OM and/or other oxidation processes can lead to the depletion of oxygen [3, 4], and the resulting anoxia is conducive to methanogenesis as the final step of OM degradation. The presence and metabolic activity of methanogenic microbes in subglacial sediments has been detected in environmental samples [5–8] and laboratory incubation experiments [9]. Methane (CH_4_) originating from microbial degradation of OM may be trapped beneath the ice and stored for extended periods of time, especially as methane hydrates [10, 11], and/or transported via the glacier drainage system and released into the atmosphere [8, 12–15]. As a potent greenhouse gas, subglacial CH_4_ may thus represent poorly constrained climate feedback. However, CH_4_ emissions can be mitigated by microbial oxidation. Under aerobic conditions, CH_4_ can be oxidized by methanotrophic microorganisms from the bacterial families Methylocystaceae and Beijerinckiaceae (Alphaproteobacteria, “type II methanotrophs”), Methylococcaceae and Crenotrichaceae (Gammaproteobacteria, “type I methanotrophs”), and Methylacidiphilaceae (Verrucomicrobia) [16]. Therefore, the amount of CH_4_ released from subglacial environments may be limited by the activity of methanotrophs [7]. Methanotrophy is a special case of methylotrophy, the ability of using reduced carbon substrates with no carbon–carbon bounds such as methanol and methylated amines [17]. Even though most methylotrophs are not capable of utilizing methane, there is evidence that they can form syntrophic relationships with methanotrophs [18, 19].

A hotspot of subglacial CH_4_ release has recently been detected in SW Greenland, with dissolved CH_4_ concentration in glacial runoff up to 1 μmol l^−1^ and air concentrations exceeding 100 ppm [12, 14]. Both types I and II methanotrophs had been detected in subglacial runoff samples in the area [6, 8, 20], and a recent comparison of microbial assemblages in proglacial rivers draining neighboring ice sheet catchments revealed a significantly higher proportion of methanotrophs in the Russell Glacier river [21] which originates at the CH_4_ hotspot site. This suggests a locally important role of CH_4_ as a substrate and, subsequently, in community assembly.

In this study, we provide a detailed description of the methylotrophic community in a glacial meltwater stream associated with the CH_4_ release hotspot in SW Greenland. We collected samples of meltwater-suspended sediment and analysed microbial assemblages using 16S rRNA gene amplicon sequencing, and place them in the context of the local hydrology and CH_4_ export.

## Methods

### Study site

**Fig. 1:**
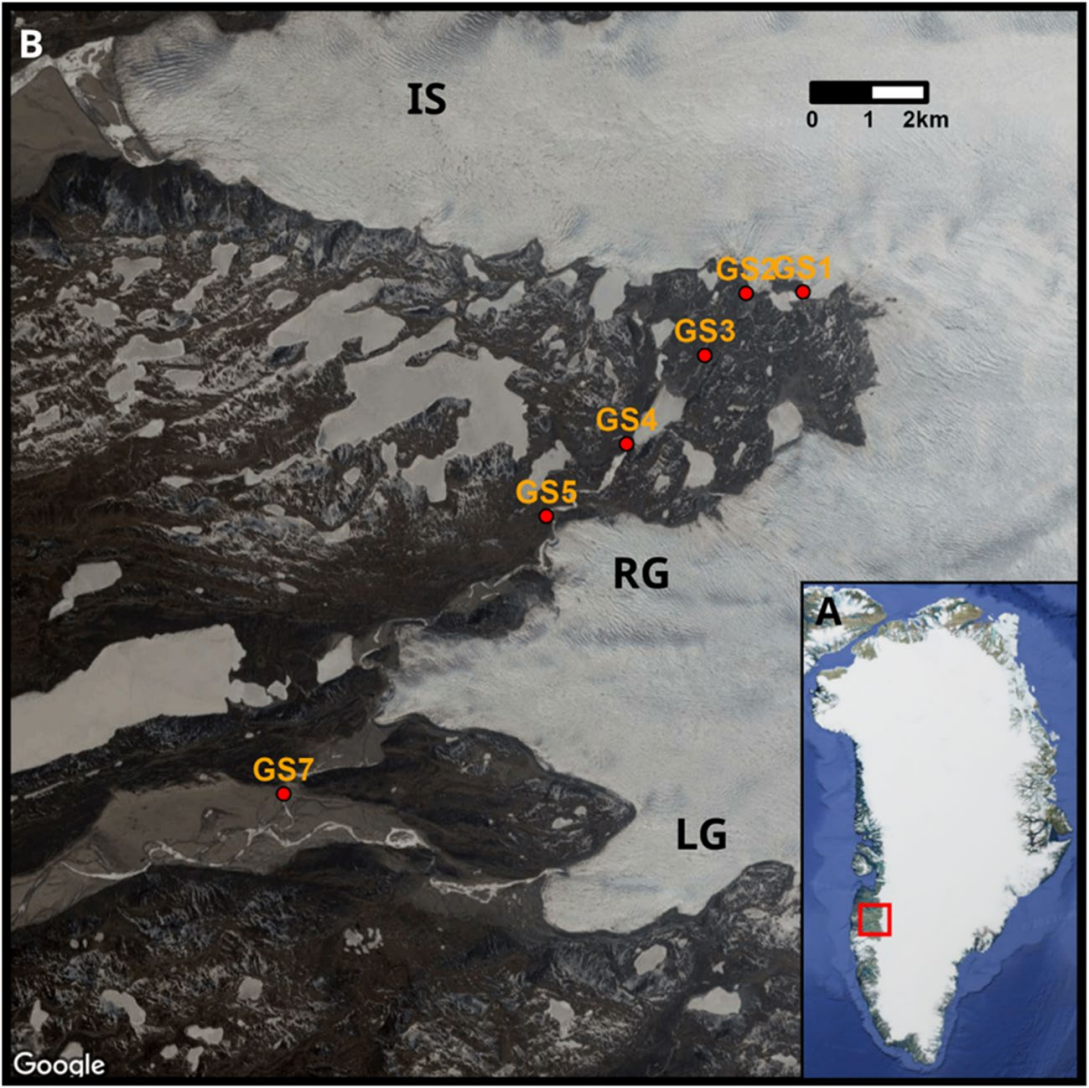
**A** Russell Glacier (RG) is located in SW Greenland. It is wedged between Isunnguata Sermia (IS) to the north and Leverett Glacier (LG) to the south. **B** Map of the sampling points of samples collected in the proglacial transect (stream transect). Samples from the portal of the RG (Portal June and Portal August) correspond to the GS1 sampling location and samples from the proglacial stream (stream June and stream July) correspond to the GS5 sampling location

Russell Glacier (RG) is a polythermal outlet glacier of the western Greenland Ice Sheet (GrIS). It is wedged between two large outlets, Isunnguata Sermia (IS) to the north and Leverett Glacier (LG) to the south (Fig. 1); its hydrological catchment is relatively small, comprising an area of approximately 81 km^2^ [21, 22]. The underlying bedrock is of metamorphic origin presumably overlain with sediment containing organic matter from numerous readvances of the ice sheet during the Holocene [23]. The Russell Glacier river originates at a portal that has been identified as a CH_4_ release hotspot [12], with air concentrations of CH_4_ exceeding 100 ppm [14], and stretches all the way along the Russell Glacier. The river then passes through several proglacial lakes [21, 24] and receives multiple glacial tributaries [25], before joining the Leverett Glacier river flowing towards Kangerlussuaq.

### Sampling

Samples of glacial meltwater were collected from the CH_4_ hotspot portal of a marginal stream coming off the RG (67.155°N, 50.069°W; ‘GS1’, Fig. 1) on 24–25 June (*n* = 6; Portal June) and 20–21 August (*n* = 7; Portal August) 2018. Concurrent measurements of CH_4_ concentrations in the air near the stream outflow were conducted and are described in Christiansen et al. [14]. Additional samples (*n* = 52; Stream June and Stream July) were collected in 2018 approximately 10 km downstream from the primary sampling site (67.104°N, 50.217°W; ‘GS5’, Fig. 1), as described by Vrbická et al. [21]. In 2019, more samples were collected along a longitudinal transect stretching from the portal site approximately 20 km downstream (*n* = 6; ‘GS1–GS7’, Fig. 1). We divided the transect samples into two groups based on their distance from the glacial portal: samples from sites GS1–GS3, i.e. before the first proglacial lake, are referred to as ‘Transect subglacial’, while samples GS4 – GS7 as ‘Transect proglacial’. Samples were taken from the water column using a sterile 50-ml syringe. Water was passed through Sterivex filters (0.22 mm; Millipore, Billerica, MA, USA) until they clogged with suspended sediment, which was between 200 and 300 ml for samples from 2018 and between 500 and 600 ml for samples from 2019. Filters were evacuated, filled with 1 mL of nucleic acid preservation buffer (LifeGuard, MO BIO, Carlsbad, CA, USA), and frozen at − 20 °C. Concurrent measurements of pH, electrical conductivity (EC), and oxygen concentration and saturation were taken using a WTW 3430 multimeter (WTW, Weilheim, Germany).

### DNA extraction

Microbial DNA was extracted using the Power Water Sterivex DNA Isolation Kit (MO BIO) according to the manufacturer’s protocol. DNA concentration was measured by using Invitrogen™ Qubit™ 4 Fluorometer with Qubit™ 1X dsDNA HS Assay Kit (Invitrogen, Carlsbad, CA, USA). The V4 region of the 16S rRNA gene was amplified by PCR using universal primers 515F (GTGYCAGCMGCCGCGGTAA) [26] and 806R (GGACTACNVGGGTWTCTAAT) [27] and sequenced on the Illumina MiSeq platform using the 2 × 250 bp arrangement at SeqMe (Dobříš, Czechia).

### Bioinformatic and statistical analysis

Raw sequences were processed using a combination of the SEED v2.1.2 [28] and DADA2 [29] pipelines. First, the paired ends of the amplicon sequences were subsequently joined using a fastq-join function [30]. After sequences join, poor-quality reads (average PHRED < 30 or length < 250 bp) were filtered out. Forward and reverse primers were cut off altogether with related tags in the program Cutadapt v4.1 [31] and the samples were rarefied to 20,000 sequences per sample in SEED. After these initial steps, the sequences were denoised, the chimeras removed, and the resulting sequences clustered into amplicon sequence variants (ASV) using DADA2. The provisional taxonomy of sequences was assigned against the SILVA v132.2 database [32]. ASVs identified as mitochondria and chloroplasts were removed from the dataset. Prokaryotes representing laboratory contaminants were identified and removed using our internal database of contaminating sequences and published lists of contaminating taxa [33, 34]. All samples were subsampled to a depth of 16,000 reads per sample. ASVs with a total abundance greater than 50 sequences were used for phylogenetic dissimilarity analysis (*n* = 758). Microorganisms potentially involved in CH_4_ cycling were preliminarily identified by comparison of their classification with known methanogenic and methylotrophic taxa. All sequences from the final dataset related to the CH_4_ cycle (i.e., methylotrophs and methanogens) were checked by BLAST using the NCBI nt/nr database. The identification of methanotrophs and methylotrophs was confirmed by phylogenetic analysis. All ASVs belonging to methano/methylotrophic clades in the phylogenetic tree were assumed to be methano/methylotrophs.

Sequences identified as methylotrophs (from ASVs with a total abundance greater than 50 sequences) were manually selected from the final dataset (*n* = 31). Homologous sequences (preferably near full length and reference if available) were searched in the NCBI database, and a total of 95 sequences were selected for the construction of the phylogenetic tree. The first BLAST hits were added to ASVs belonging to yet undescribed microorganisms. Obtained sequences were aligned in MAFFT [35] using the method G-INS-i (other settings options were set as default). Nonaligned parts of sequences were manually removed in BioEdit [36]. The maximum likelihood phylogenetic tree was generated in raxmlGUI2.0 [37], a graphical interface to RAxML [38]. The number of runs was set at 50, and the model used was GTRGAMMA. Branch support was assessed with aBayes in PhyML v3.0 [39]. The tree was visualised with TreeView [40].

Statistical analysis and data visualisation were performed using R v4.2.1. As a metric of alpha diversity, the Shannon diversity index was calculated using the microeco package v0.11.0 [41]. Map of the sampling locations was created with the ggmap package v3.0.1 [42]. The significance of differences in diversity and richness between samples from sampling locations was tested using the Dunn’s Kruskal–Wallis test. Indicator species analysis was performed to determine which ASVs significantly differed in relative abundance between groups. This analysis was done with the package indicspecies v1.7.12 [43]. To calculate the phylogenetic beta diversity measures, the top 758 ASVs (ASVs with higher absolute abundance than 50 sequences) were aligned in MAFFT, trimmed in BioEdit, and a phylogenetic tree was constructed in RAxML. Subsequently, the weighted Unifrac distances were calculated in Mothur [44] for the whole community and ASVs identified as methylotrophs. Principal coordinate analysis (PCoA) was performed to visualise differences in composition between sample groups. The significance of the sampling month and location on the structure of the microbial assemblage was tested by analysis of molecular variance (AMOVA) in Mothur.

## Results

### Methane export

**Fig. 2:**
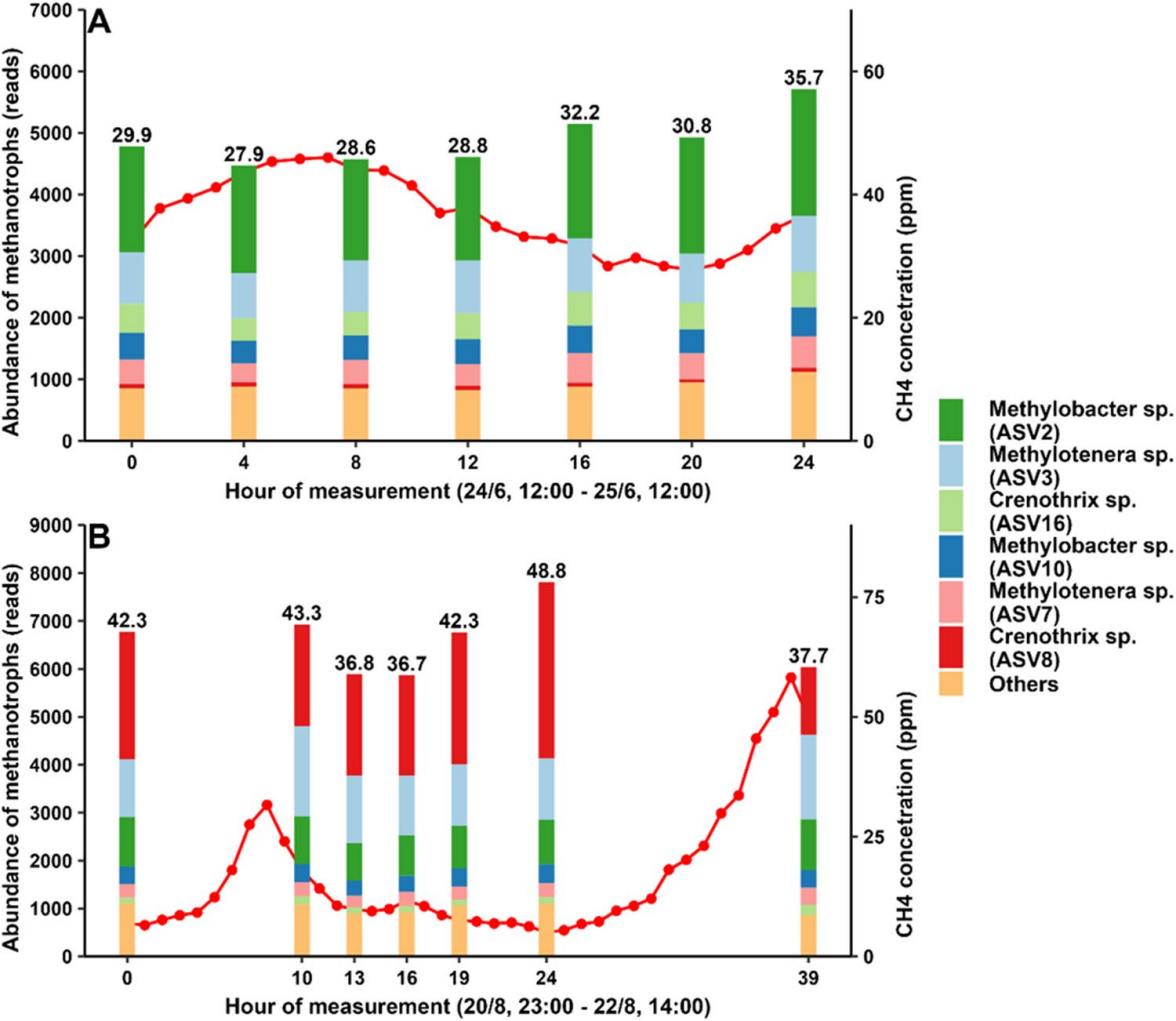
Changes in the abundance of top 6 methylotrophs from subglacial portal samples within sampling days in June (**A**) and August (**B**). Red lines correspond to CH_4_ concentration measured in the air at ‘GS1’. The average concentration of CH_4_ in June was 38.7 ppm (min = 27.9 ppm, max = 53.4 ppm). The average concentration of CH_4_ in August was 19.2 ppm (min = 5.1 ppm, max = 58.2 ppm) [14]. The number above the column shows the relative abundance of methanotrophs in %

The average concentration of CH_4_ in the air at the portal in June (GS1) was 38.7 ppm and ranged from 27.9 to 53.4 ppm (Fig. 2). The pH of the meltwater was 7.19, electrical conductivity (EC) was 10.6 μS cm^−1^ and the oxygen concentration (O_2_) was 14.1 mg l^−1^, corresponding to a saturation of 102.5%. The concentration of CH_4_ at the portal in August (GS1) showed more fluctuation. The average concentration was 19.2 ppm and ranged from 5.1 ppm to 58.2 ppm (Fig. 2). The isotopic signature of CH_4_ indicated that it likely originated from microbial methanogenesis and that its source was stable through the season [14].

### Exported assemblages

**Fig. 3:**
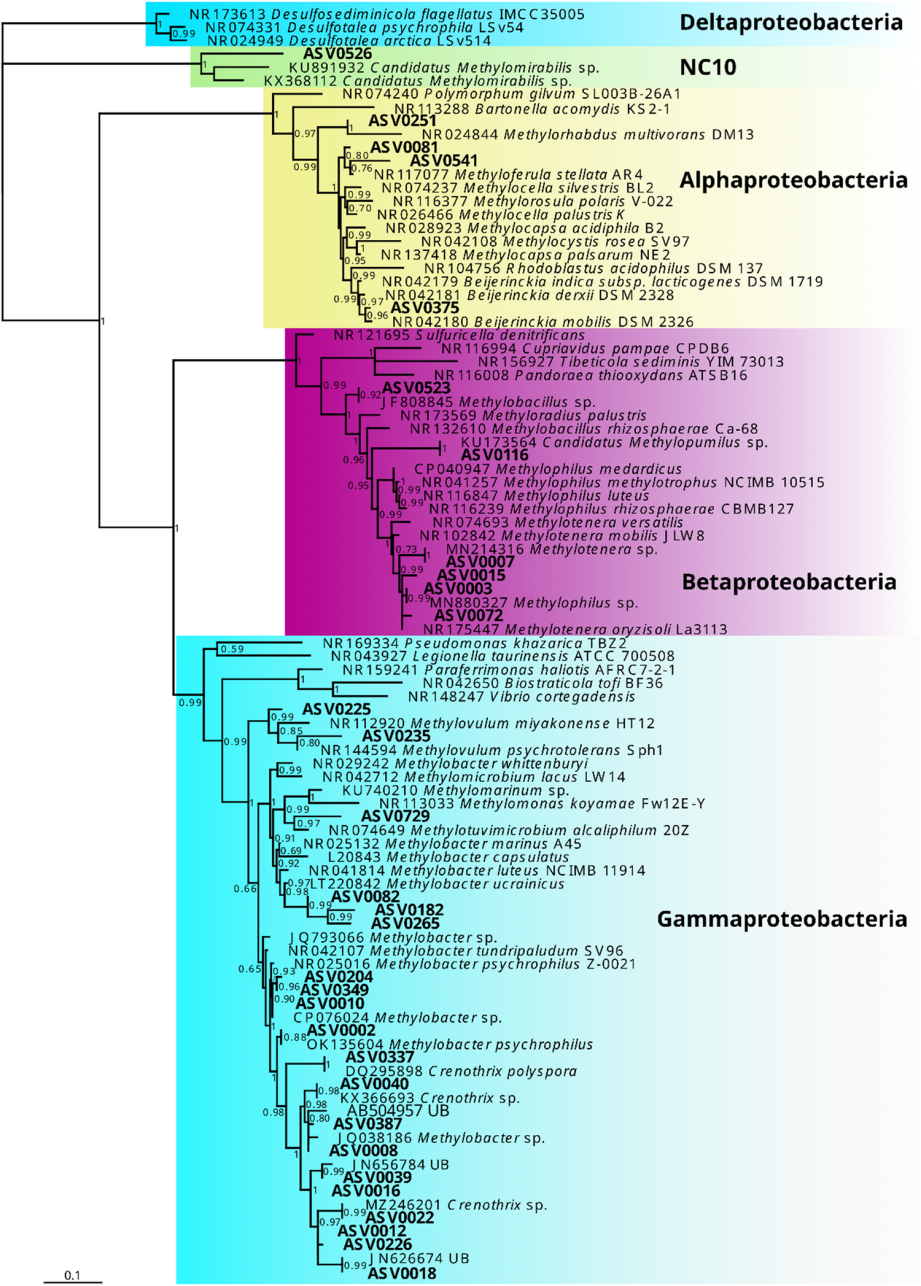
Phylogenetic tree of methylotrophs detected in collected meltwater assemblages (in bold). From 31 ASVs identified as methylotrophs, 20 ASVs clustered within Crenotrichaceae and Methylococcaceae (Gammaproteobacteria), six within Methylophilaceae (Betaproteobacteria), four within Beijerinckiaceae (Alphaproteobacteria), and one sequence (ASV0526) was related to the candidate phylum NC10. aBayes values > 0.5 are shown at nodes

Exported assemblages were dominated by Proteobacteria, followed by Bacteroidetes and Actinobacteria. (Supplementary Table 2). Thirty-one ASVs were identified as methylotrophs, contributing with 27.2% of total reads to the analysed assemblages. The constructed phylogenetic tree revealed that twenty ASVs (17.5% of total reads) clustered within the groups Crenotrichaceae and Methylococcaceae (Gammaproteobacteria; type I methanotrophs), six (9.4% of total reads) within the Methylophilaceae (Betaproteobacteria), four (0.3% of total reads) within the Beijerinckiaceae (Alphaproteobacteria; type II methanotrophs or non-CH_4_ consuming methylotrophs), and one sequence (ASV0526) was related to the candidate phylum NC10 (Fig. 3). Archaeal ASVs accounted for less than 0.5% of all sequence reads and were mostly represented by Euryarcheota and Thaumarcheota. The most abundant archaeal ASV was identified as the methanogen *Methanoregula* sp., with a mean relative abundance lower than 0.05%. For most ASVs, it was not possible to determine their taxonomy on a finer scale even after phylogenetic tree construction since there were not enough relevant taxonomically identified sequences available in the database.

No significant correlation was found between the abundance of CH_4_-related (methanogenic or methanotrophic) ASVs and the concentration of CH_4_ in the air at the portal.

### Temporal changes in exported methylotrophic assemblages

**Fig. 4:**
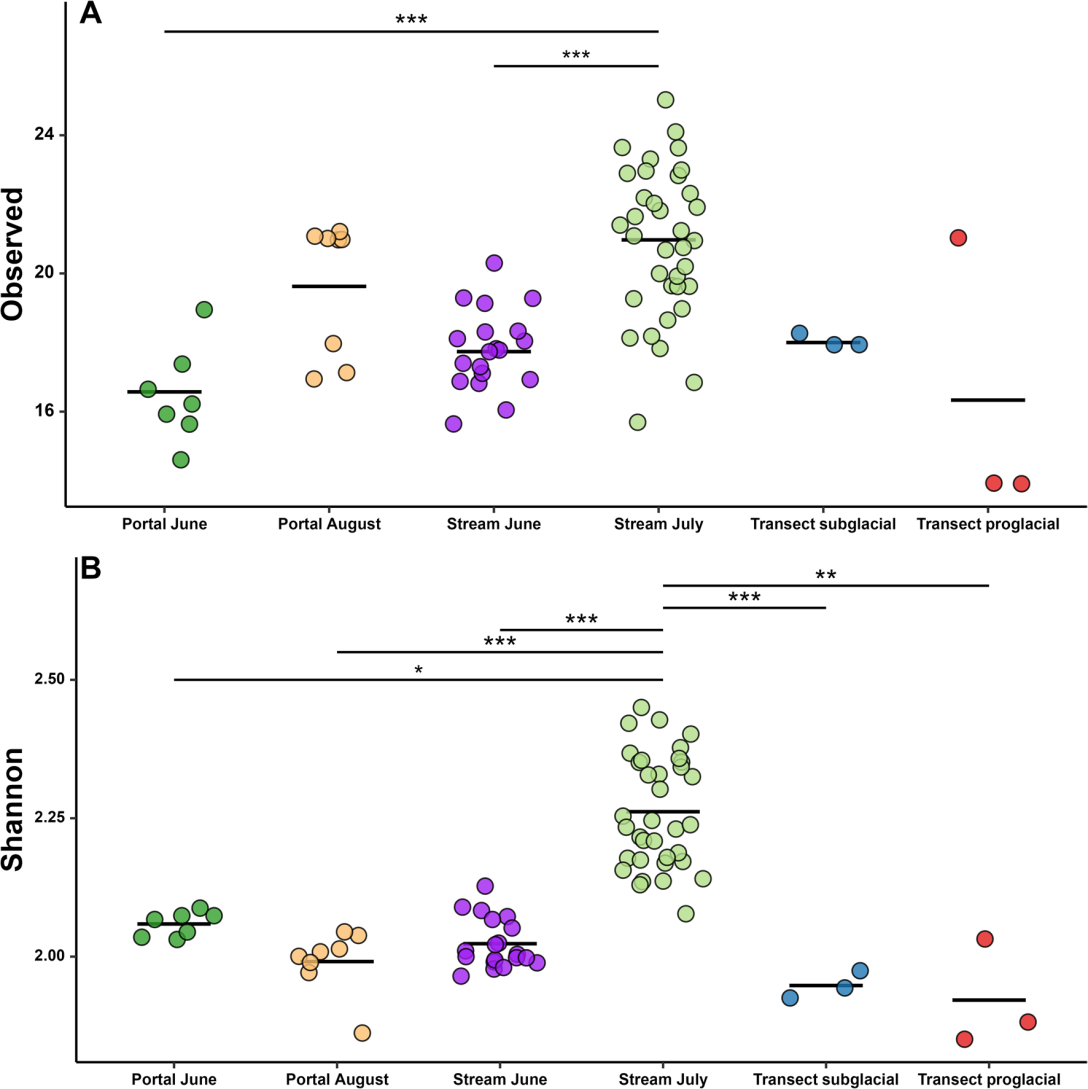
Observed richness (**A**) and Shannon diversity index (**B**) of methylotrophs for all sampling locations. The highest richness and diversity were measured in stream July samples. The significance of the differences in richness and Shannon diversity was tested with Dunn’s Kruskal–Wallis test. Asterisks show the level of significance (*p < 0.05; **p < 0.01; ***p < 0.001). Line within the sample column indicates the mean value

**Fig. 5:**
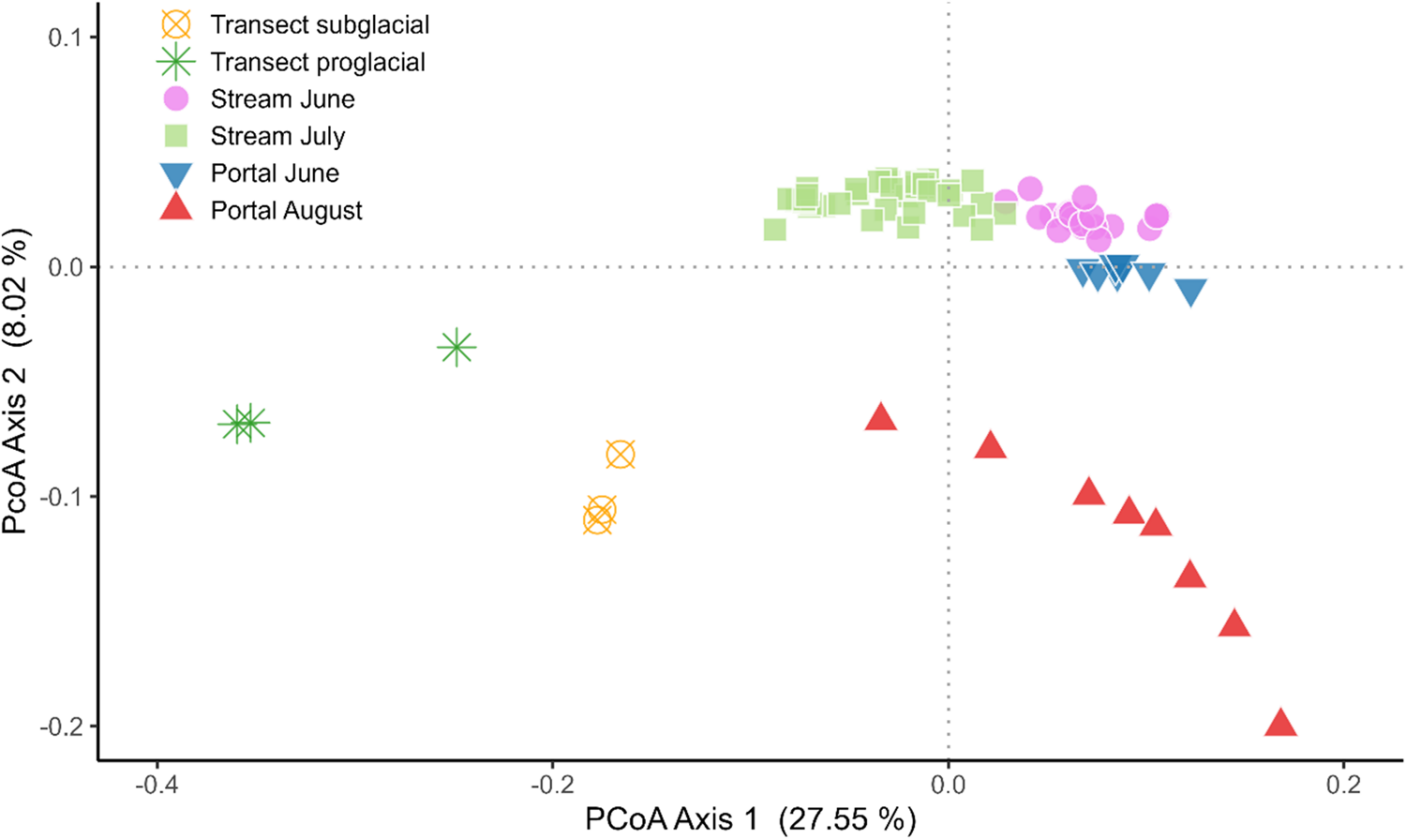
PCoA showing clustering of samples of methylotrophs using weighted UniFrac distance matrix

Twenty-eight ASVs of methylotrophs were found in the samples collected at the portal site (Portal June and Portal August). Twenty ASVs were shared between both months and only two and six were unique to June and August, respectively. The observed richness was higher in the Portal August samples (mean = 19.6) than in the Portal June samples (mean = 16.6). Shannon’s diversity index was found to be slightly higher in the Portal June samples (mean = 2.1) than in the Portal August samples (mean = 2.0); however, these differences were not statistically significant (Dunn’s Kruskal–Wallis test; *p* > 0.05) (Fig. 4). When plotted as PCoA using weighted UniFrac (Fig. 5), methylotrophs from June and August clustered separately and the clustering was found significant (AMOVA; *p* < 0.001).

The relative abundance of methylotroph related sequences was found to be different between the 2 months. On average, 30.6% of the reads in the June samples were identified as methylotrophs, while in the August samples it was 41.1% (Fig. 2). The composition of the methylotrophic assemblage also differed between months. For example, the relative abundance of ASV0008 was more than ten times higher in August in comparison with June. Overall, ASVs ASV0008, ASV0003, ASV0018, and ASV0072 were found to be associated with the August samples, while ASV0002, ASV0016, and ASV0012 were found to be associated with the June samples (indicator species analysis; *p* < 0.001).

### Longitudinal changes

To determine if there were any changes in the composition of methylotrophs as they travelled downstream, the composition of exported methylotrophs from the portal samples (Portal June, Portal August) was compared with methylotrophs found in the proglacial samples from the seasons 2018 (stream June, stream July) and in the transect samples from 2019 (transect subglacial, transect proglacial). Altogether, 31 ASVs were identified as methylotrophs. Fifteen of them (90.5% of methylotrophic sequences) were found in all samples. Two (0.04% of methylotrophic sequences) methylotrophic ASVs were unique to the portal samples (ASV0349 and ASV0387; both from Gammaproteobacteria). Three (2.9% of methylotrophic sequences) methylotrophic ASVs were found only in the samples farther downstream (stream June, stream July, and transect proglacial) (ASV0081, ASV0116, and ASV0251; all of them from Betaproteobacteria and Alphaproteobacteria). The rest of the methylotrophic ASVs were present in multiple sampling locations, but not in all of them. Differences in alpha diversity were also found (Fig. 4). Methylotrophs had the highest diversity (mean = 2.3) and richness (mean = 20.9) in the proglacial samples from July 2018 (Stream July) and the lowest diversity (mean = 1.9) and richness (mean = 16) in the transect samples further downstream from 2019 (Transect proglacial). Shannon’s diversity index was found to be significantly different between the proglacial samples from July and all other sampling locations (Dunn’s Kruskal–Wallis test; *p* < 0.05). However, no significant differences in diversity were found between the rest of the sampling locations. Richness was found to be significantly different between the samples from Stream July, Portal June, and Stream June (Dunn’s Kruskal–Wallis test; *p* < 0.05).

When comparing only methylotrophs, the portal samples from June (portal June) clustered close to the proglacial samples from June (stream June) and July (stream July), while the portal samples from August (portal August), the subglacial transect samples (transect subglacial) and the proglacial transect samples (Transect proglacial) clustered separately (Fig. 5). However, all these clusters were found to be statistically significant (AMOVA; *p* < 0.05). We found that samples from different sampling locations clustered separately not only for methylotrophic ASVs but also when the whole assemblages were assessed (Supplementary Fig. 6).

The composition of exported methylotrophic assemblages differed not only in season but also as they travelled downstream. On average 29.6% of reads in the transect subglacial samples were identified as methylotrophs while in the Proglacial transect samples it was only 12.9% of reads (Supplementary Table 3). Methylotrophs related to the type II methanotrophs were more abundant in the transect proglacial samples than in the transect subglacial samples (Supplementary Table 3, Fig. 7).

## Discussion

### Exported assemblages

At higher taxonomic levels, the composition of exported microbial assemblages from the CH_4_ release hotspot site was comparable to those previously found in subglacial meltwaters [20, 45–47] and sediments [2, 48, 49]. However, in contrast to most previous studies, we observed a remarkable prevalence of ASVs affiliated with methylotrophic genera. This agrees with a previous study from the same glacier which reported high relative abundances of methanotrophs reaching up to 66% [6]. However, contrary to our findings the previous study reported lower diversity of exported methanotrophs and the dominance only of one exported methanotroph (identified as *Methylobacter psychrophilus*) over the course of a melt season. The high relative abundance of methylotrophs is not surprising considering the high local CH_4_ emissions [12, 14], which serve as a substrate for methanotrophs. Furthermore, this phenomenon is already known from other parts of the cryosphere such as permafrost soils [50, 51] and Arctic lakes [52], where the emissions of CH_4_ are high.

The most abundant ASV (ASV0002) in the methylotrophic assemblage was identified as closely related to *Methylobacter psychrophilus*, the second most abundant methanotroph (ASV0008, the fourth most abundant ASV) was found to belong to an uncultured group related to *Crenothrix polyspora* (Table 1, Fig. 3). We found that the majority of the methanotrophs detected were ‘type I’, i.e., members of Gammaproteobacteria (20 ASVs; 17.5% of all reads), while methanotrophs from Alphaproteobacteria (“type II”) were represented at substantially lower abundances (2 ASVs; 0.2% of all reads). A possible explanation for the dominance of type I methanotrophs lies in the different physiological requirements of type I and II methanotrophs (see below).

**Table 1:** List of methylotrophic ASVs whose relative abundance exceeded 1% in at least one sample

ASV	RA [%]	GB acc	cov [%]	id [%]	The closest BLAST hit
ASV0002	8.2	OK135604	100	100	*Methylobacter psychrophilus*
ASV0003	5.2	NR_041257	100	99.6	*Methylophilus methylotrophus*
ASV0007	2.4	NR_175447	100	98.81	*Methylotenera oryzisoli*
ASV0008	2.0	KX366693	100	99.6	*Crenothrix* sp.
ASV0010	1.8	NR_025016	100	99.6	*Methylobacter psychrophilus*
ASV0012	1.5	NR_112920	100	96.44	*Methylovulum miyakonense*
ASV0015	1.3	NR_175447	100	98.80	*Methylotenera oryzisoli*
ASV0016	1.1	MZ246201	100	98.81	*Crenothrix* sp.

*RA* Mean relative abundance in the whole dataset; *GB acc.* accession number in GeneBank; *cov* query coverage; *id* percent identity

Surprisingly, we found that other highly abundant ASVs (ASV0003 and ASV0007; the second and third most abundant methylotrophic ASV, respectively) were identified as methylotrophs of the *Methylotenera* and *Methylophilus* (Betaproteobacteria) (Table 1, Fig. 3), which are probably not capable of metabolizing CH_4_ [17]. We offer three possible explanations for this finding. First, it could be explained by in situ syntrophic interaction between the methanotrophs and non-CH4 consuming methylotrophs. It has been suggested that a few intermediates of CH_4_ oxidation (e.g., formaldehyde and methanol) can be exuded during methanotrophy and utilised by methylotrophs from the Betaproteobacteria [53, 54]. Furthermore, van Grinsven et al. [19] have shown that the transfer of CH_4_-derived carbon from the methanotroph *Methylobacter* to methylotroph *Methylotenera* can occur in laboratory incubation experiment using ^13^C-labeled CH_4_. Second, it may be the result of the passive mixing of microbes from different sources in the meltwater stream (i.e., subglacial sediment, supraglacial meltwater). In a recent study, members of the Methylophilaceae were found to be abundant in samples from subglacial sediments [2], where they may utilize intermediate products of metabolism of various heterotrophic bacteria. Therefore, these methylotrophs may be sourced from subglacial sediment where methylated substrates may be found as products of various OM degradation processes. Third, it is possible that the identified ASVs may be able to utilise CH_4_ after all. This question certainly warrants further attention.

### Temporal changes in exported methylotrophic assemblages

Despite the distinct clustering of the portal samples from June and August in the PCoA (Fig. 5), a substantial part of the exported ASVs was shared by both. The differences were thus mainly driven by the ASVs’ abundances. This is probably due to the passive mixing of assemblages from various glacier sources (i.e., supraglacial, subglacial) that are interconnected through the glacier drainage system. Because of the seasonal development of the drainage system, this connection is dynamic and may result in a different mixing contribution during the melting season [20, 46, 47].

The two most abundant methanotrophic ASVs (ASV0002 and ASV0008) were present in both portal assemblages from June and August (Portal June and Portal August); however, their relative abundances differed by more than an order of magnitude between these two months. The finding that ASV0008 is related to *Crenothrix*, a filamentous bacterium [55] suggests an alternative explanation of this difference, namely that it may be the result of environmental selection for taxa capable of resisting rheological stress (increased meltwater flow velocity later in the melt season) at the expense of the non-filamentous *Methylobacter* (ASV002). Those ASVs affiliated with *Crenothrix* may remain in the hydrologically stressful yet substrate-rich environment for longer periods and so grow to higher abundances which are then reflected in the exported assemblages later in the melt season.

On the other hand, the overall higher diversity and richness in the August samples could also be explained by the seasonal development of the drainage system. As a result of the higher interconnectedness of the glacial environments, microorganisms are sourced not only from the inner part of the subglacial environment but also from larger portions of the supraglacial environment, as previously described [45, 46]. However, the main source(s) of methylotrophs for the meltwater assemblages remain uncertain.

### Longitudinal changes

As the distance from the ice sheet margin increased, the relative abundance of sequences identified as methylotrophs decreased. Furthermore, the relative abundance of ASVs related to the alphaproteobacterial methanotrophs increased, compared to those of Gammaproteobacteria (Supplementary Table 3). This is probably due to the increased contribution of methylotrophs originating in pro- and periglacial environments (e.g., soils and proglacial lakes) to the transported assemblages. The different physiological requirements of type I and type II methanotrophs may play a role, and the lack of direct CH_4_ emissions further downstream could explain why these two groups of methanotrophs contribute differently to the transported assemblages at different locations. Type I methanotrophs have been shown to thrive at lower temperatures [6] and when oxygen concentration is low and CH_4_ concentration is high [56] which is typical of the conditions in the subglacial environment. For example, in a study from Arctic permafrost, it was found that *Methylobacter*-related species were more abundant in places with high CH_4_ emissions (thawed fen), while alphaproteobacterial methanotrophs were more abundant in intact palsa with lower CH_4_ emissions [51]. Since the conditions in the subglacial environment are rather limited by oxygen, it is possible that type I outcompete type II methanotrophs there. Furthermore, type I methanotrophs were recently found to be active in the anoxic sediments of an Arctic lake [57], suggesting that gammaproteobacterial methanotrophs in our samples could be sourced even from the subglacial sediments farther inward.

Unfortunately, 16S rRNA gene amplicon data do not provide information about the activity of the microorganisms detected, and so it is not possible to infer whether the methylotrophs found in the proglacial samples were actively consuming CH_4_ and/or other C_1_ substrates in situ. Previously, microorganisms originating from the subglacial environment were shown to potentially reactivate after deposition in estuary sediments [58], however, further research is necessary to confirm this assumption.

## Conclusion

Methylotrophs made up approximately 30% of exported microbial assemblages at a subglacial CH_4_ export hotspot in SW Greenland. They were dominated by Methylococcaceae and Crenotrichaceae (Gammaproteobacteria; type I methanotrophs), followed by Methylophilaceae (Betaproteobacteria; non-CH_4_ consuming methylotrophs). No correlation between the relative abundance and/or diversity of CH4-related microorganisms (methanogens and methylotrophs) and CH_4_ concentration in the air at the portal was found. Differences in the composition of exported assemblages were detected both between June and August and along a longitudinal stream transect, possibly due to the combination of glacial drainage system development and non-glacial inputs further downstream. Our results suggest that sites with significant subglacial methane release can be colonized by microorganisms that can potentially reduce the methane emissions.

### Supplementary Information

Below is the link to the electronic supplementary material.

Supplementary file1 (XLSX 21 KB)

Supplementary file2 (PDF 303 KB)

## Data Availability

The 16S rRNA amplicon sequencing dataset of the samples newly sequenced in this study is available at the NCBI under the accession number PRJNA936828. Data for the rest of the samples were sourced from Vrbická et al. [21].
