# Supplementary material for: Methylotrophic Communities Associated with a Greenland Ice Sheet Methane Release Hotspot
Source: Microb Ecol. 2023 Oct 16;86(4):3057–67. doi: 10.1007/s00248-023-02302-x (PMC10640400; doi:10.1007/s00248-023-02302-x)
Supplement: Supplementary file 2 — Supplementary file2 (PDF 303 KB) [file 248_2023_2302_MOESM2_ESM.pdf]

Methylotrophic communities associated with a Greenland Ice Sheet methane release hotspot

Matěj Znamínko[1,*], Lukáš Falteisek[1], Kristýna Vrbická[1], Petra Klímová[1], Jesper R. Christiansen[2], Christian J. Jørgensen[3], Marek Stibal[1]

[1]*Department of Ecology, Faculty of Science, Charles University, Prague, Czechia*

[2]*Department of Geoscience and Natural Resource Management, University of Copenhagen, Copenhagen, Denmark*

[3]*Department of Ecoscience, Arctic Environment, Aarhus University, Roskilde, Denmark*

**current address: Department of Environmental and Biological Sciences, University of Eastern Finland, Kuopio, Finland*

Keywords: methylotrophs, methanotrophs, subglacial environment, Greenland Ice Sheet

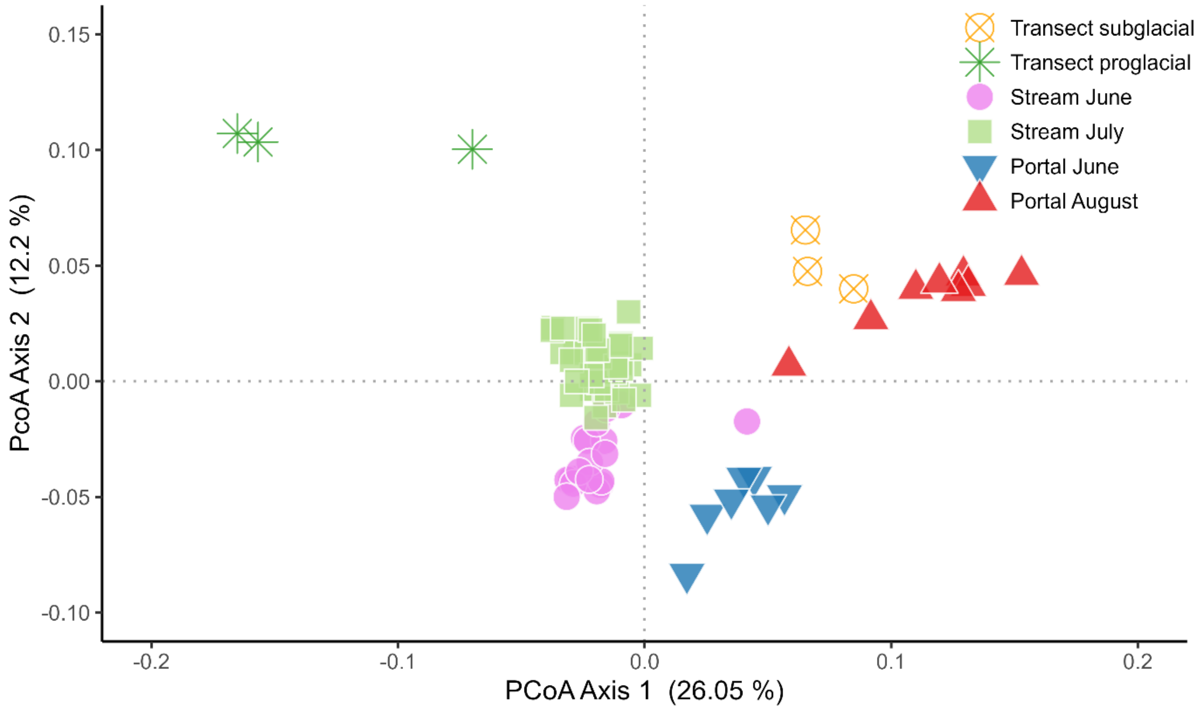

Figure 6 PCoA showing clustering of samples based on the whole community using weighted UniFrac distance matrix.

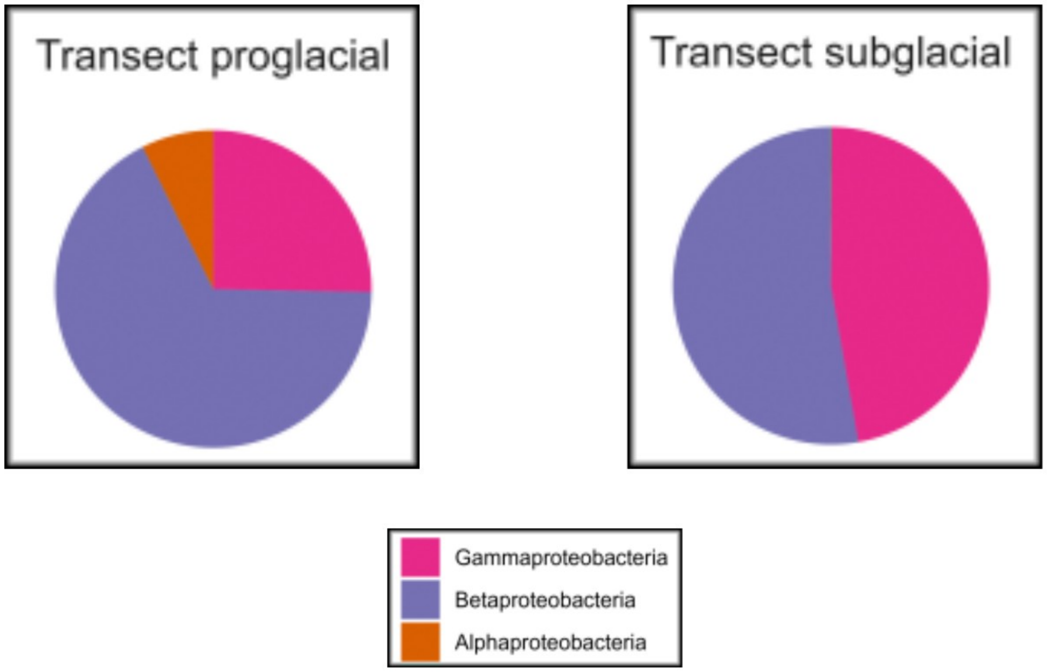

Figure 7 Changes in the composition of exported methylotrophic groups between Transect subglacial and Transect proglacial samples.
